# Functional outcomes of simultaneous anterior cruciate ligament reconstruction and lateral extra-articular tenodesis using an all-suture anchor: a modified mini-open technique

**DOI:** 10.20452/wiitm.2025.17938

**Published:** 2025-03-12

**Authors:** Jakub Erdmann, Maria Zabrzyńska, Przemysław Pękala, Szymon Nowak, Filip Gołębiewski, Gazi Huri, Jan Zabrzyński

**Affiliations:** Department of Orthopaedics and Traumatology, Faculty of Medicine, Collegium Medicum in Bydgoszcz, Nicolaus Copernicus University in Torun, Bydgoszcz, Poland; Department of Family Medicine, Collegium Medicum, Nicolaus Copernicus University in Torun, Toruń, Poland; International Evidence‑Based Anatomy Working Group, Department of Anatomy, Jagiellonian University Medical College, Kraków, Poland; Department of Orthopaedics and Traumatology, Hacettepe University School of Medicine, Ankara, Poland; Department of Orthopaedic and Sports Medicine, Hospital Doha, Doha, Qatar

**Keywords:** all‑suture anchor, anterior cruciate ligament reconstruction, lateral extra‑articular tenodesis, Lemaire technique, mini‑invasive

## Abstract

**INTRODUCTION:**

The anterior cruciate ligament (ACL) rupture frequently leads to instability of the knee joint, which subsequently damages other intra‑articular structures. The combination of ACL reconstruction (ACLR) with concurrent lateral extra‑articular tenodesis (LET) improves rotational stability and reduces the risk of subsequent ACL rupture. However, there is not much research that specifically outlines LET hardware and surgical methods.

**AIM:**

This study aimed to describe and evaluate clinical outcomes of a mini‑open modified Lemaire technique using a self‑punching all‑suture anchor.

**MATERIALS AND METHODS:**

In this study, 32 patients underwent primary or revision ACLR combined with LET via the mini‑open modified Lemaire technique using a self‑punching all‑suture anchor. All individuals completed the following pre‑ and postoperative questionnaires to evaluate their functional performance: the Knee Injury and Osteoarthritis Outcome Score, assessing several domains, the International Knee Documentation Committee subjective knee evaluation form, the Lysholm knee scoring scale, and the Western Ontario and McMaster Universities Arthritis Index. Complication rates were also assessed.

**RESULTS:**

Each patient’s functional score values increased, as compared with preoperative measure‑ ments. There were no early post‑ or intraoperative complications associated with the technique described.

**CONCLUSIONS:**

This is the first study that evaluated clinical outcomes, intraoperative, and early post‑ operative complications of the mini‑open modified Lemaire technique using a self‑punching all‑suture anchor. Our study indicates that this procedure is effective, safe, and associated with better cosmesis than classic LET techniques.

## INTRODUCTION 

In the United States, it is estimated that over 120000 anterior cruciate ligament (ACL) injuries occur each year, mostly among high school and college-age individuals.^1^ ACL rupture may lead to persistent knee instability and the risk of subsequent chondral lesions, meniscal injuries, and early osteoarthritis.^2^ ACL reconstruction (ACLR) is recommended to mitigate these effects, especially in young, active patients.^3^ Despite the fact that ACLR successfully decreases anterior tibial translation, 25%–30% of patients experience postoperative rotational instability, and the ACL graft failure rate remains comparatively high (17.1%–18%).^4^^,^^5^ Thus, ACLR has been recently combined with lateral extra-articular tenodesis (LET) procedures or anterolateral ligament (ALL) reconstructions that reliably decrease rotational knee instability and reduce the risk of ACL graft failure.^6^^,^^7^ It was found that patients after ACLR with concurrent LET/ALL reconstruction are 2-to-4 times less likely to experience graft failure than individuals after isolated ACLR.8 According to biomechanical research, LET aids in offloading the ACL graft, which reduces anterior tibial translation in response to rotational forces.^9^

**FIGURE 1:**
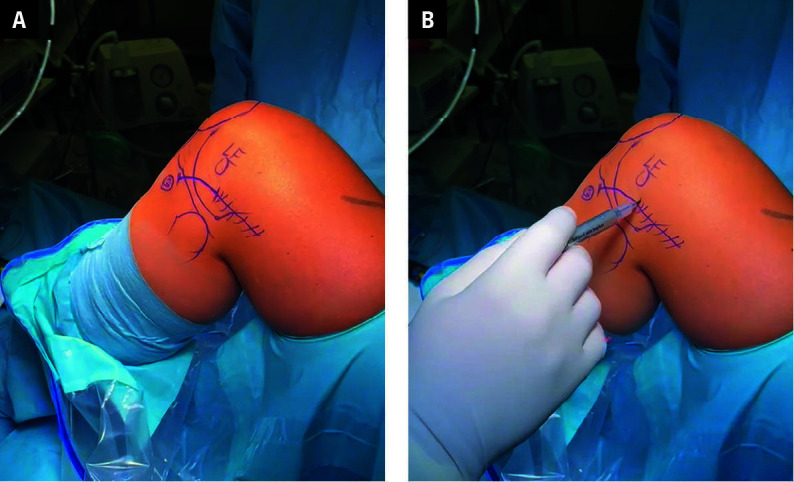
Structures such as the lateral epicondyle (LE), fibular head, patella, and Gerdy tubercle were palpated and marked with a marking pen. The surgical incisions of the mini‑ ‑open (solid line) and classic lateral extra‑articular tenodesis (dashed line) were marked

The aforementioned data led to an increase in literature supporting the use of the abovementioned methods during ACLR, especially among patients with high-grade pivot shift, knee laxity, concomitant meniscal lesions, revision ACLR, and in physically active groups.^8^^,^^10^ However, these indications are not definitive and remain a subject of debate. The LET technique was first described by Lemaire in 1967.^11^ Recently, it has garnered increasing interest, which has spurred the development of numerous new surgical techniques.^12^ The Lemaire technique and its modified form, as well as the Ellison, MacIntosh, and Arnold–Cocker techniques, rely on the use of autografts or allografts to strengthen the knee’s lateral structures.^9^ The majority of them harvest a strip of the iliotibial band (ITB) and pass it under the lateral collateral ligament (LCL), which ultimately improves anterior laxity and tibial internal rotation.^13^ The optimal area of graft implementation and the method of its fixation, including the used device, differ across the techniques. However, the literature comparing clinical outcomes of particular methods is limited. For instance, initial studies do not show significant differences in patient outcomes between the modified Lemaire and MacIntosh procedures.^14^ Although there are many surgical techniques describing the implementation of LET, the modified Lemaire procedure seems to be widely favored.^15^

## AIM

The aim of this study was to describe a new mini-open LET approach that uses a self-punching all-suture anchor. It is an extension of the modified Lemaire technique and is employed in addition to ACLR in a group of selected patients who require greater control over their internal tibial rotation. Moreover, this study also aimed to assess clinical outcomes and complications after the aforementioned procedure.

## MATERIAL AND METHODS

General data This study was conducted at the Department of Orthopaedic Surgery between March 2023 and November 2024, and was approved by the local bioethics committee (KB 347/2023). It was designed as a retrospective study of the short-term outcomes of ACLR with LET using a novel mini-open technique with an all-suture anchor. All participants gave an informed consent before undergoing the procedures.

The present study included 32 patients who underwent primary or revision arthroscopic ACLR with LET, using the modified Lemaire technique. All patients presented with chronic (>3 weeks), post-trauma instability and pain in the affected knee. An ACL tear on noncontrast knee magnetic resonance imaging (MRI) and physical examination (anterior drawer test, pivot shift test, and the Lachman test) were used to preoperatively confirm the instability in each case.

The inclusion criteria comprised a complete ACL tear with symptomatic instability, age of 18 to 64 years, and patient expectations of returning to the preinjury level of sports performance. Patients were excluded if they had under- gone a prior surgery on the contralateral knee or presented with an ACL tear without symptomatic instability, a contralateral knee injury, significant malalignment, or a multiligament knee in- jury, or if their age was below 18 years or greater than or equal to 65 years.

All preoperative evaluations and operations were performed by a senior orthopedic surgeon (JZ) who was experienced in knee arthroscopy and ACLR, and performed LET routinely (more than 50 cases per year).

Similar procedures for the surgical treatment of isolated ACLR and the rehabilitation process are described in a study by Szwedowski et al,^16^ whose research team included one of the authors of this publication.

### Operative technique

The surgery was performed under general or intraspinal anesthesia with the patient placed in the supine position. The affected knee was evaluated to confirm that the observed instability was consistent with ACL insufficiency and MRI findings. The pivot shift test with verification of the excessive internal tibial rotation was carried out in each case. Around the affected leg’s thigh, a tourniquet and a leg holder were applied. The knee was placed at 90 degrees of flexion, and structures such as the lateral epicondyle, fibular head, patella, LET approach, and Gerdy tubercle were palpated and marked with a sterile marking pen FIGURE 1. Standard anterolateral and anteromedial knee portals were used. Diagnostic arthroscopy was per- formed to evaluate abnormal findings, primarily to confirm a complete ACL rupture. Concomitant lesions, such as medial and lateral meniscal tears, were trimmed to a stable rim. Cartilage lesions were probed, measured, and then graded during surgery according to the Outerbridge classification. Intraoperative findings were collected in a standardized format.

**FIGURE 2:**
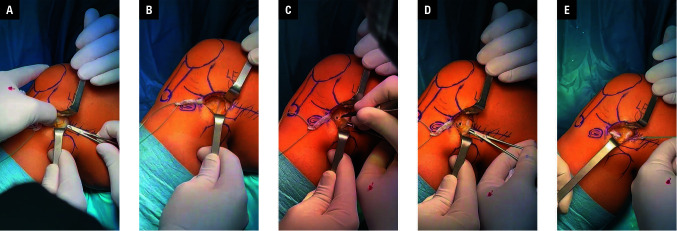
Intraoperative images showing the initial stages of lateral extra‑articular tenodesis; A – placement of a vascular clamp under the lateral collateral ligament (LCL) to prepare a tunnel for the iliotibial band (ITB) passage; B – securing of the ITB graft using whip stitches; C – performing the anterior entrance to the tunnel underneath the LCL without a 15 surgical blade; D – introduction of a vascular clamp further to the exit, in front of the LCL; E – passing the ITB graft under the LCL and grasping the stitches with the clamp

After arthroscopic assessment, which involved confirming a complete ACL tear and address- ing additional intra-articular injuries, the ACLR was initiated. The standard ACLR procedure was carried out according to Siebold’s guidelines.^17^ An autologous 4-strand hamstring graft (semi-tendinosus and gracilis [STG] tendons) was per- formed in each primary ACLR, while a bone-tendon-bone (BTB) autograft was applied in each revision ACLR. Since a majority of the participants underwent primary ACLR, the focus was limited to the STG graft procedure. Proxi- mally and medially to the insertion of the pes anserinus, a 3–4 cm oblique incision was made. The STG tendons were identified and the associ- ated fascial bands were meticulously dissected. They were harvested using an open-ended ten- don stripper. To obtain better visualization and precise anatomy of the LET, in this method, it is advised to continue with the LET technique and harvest the ITB before these structures become swollen by arthroscopy fluid.

The obtained tendons were prepared to re- move any remaining muscle tissue. They were then secured in a looped configuration using the Ethibond Excel polyester suture (Ethicon, Raritan, New Jersey, United States) to create a double-folded graft. Subsequently, the graft was measured with a sizer, and the femoral tunnel was drilled correspondingly to the relevant size. The femoral point of entry was marked before drilling in compliance with the antero- medial technique and under arthroscopic guidance. Next, the tibial tunnel was drilled in a routine fashion using an ACL elbow guide. When the tunnels were prepared, the Endobutton system (Infinity Femoral Adjustable Loop Button, CONMED, Utica, New York, United States) was loaded with the graft and subsequently pulled through the canals, flipped outside, and anchored to the femoral cortex. The sufficiency of the ACL graft and knee range of motion were checked arthroscopically. With the knee at 30 de- grees of flexion and maximum stretch on the dis- tal part of the graft, it was fixated using an in- terference screw with a diameter corresponding to the previously measured graft size (GENESYS Matryx Interference Screw, CONMED).

Then, the knee was set in the 90-degree flexed position and in neutral rotation. Mini-open LET, based on the modified Lemaire technique, was performed with a reduced 3–4 cm skin incision between the lateral femoral epicondyle and Gerdy tubercle. Dissection was performed to identify and expose the ITB. A 1 cm × 8 cm ITB strip was harvested along the axis of the ITB to- ward the lateral femoral condyle FIGURE 2. Distal insertion of the ITB was persevered, while the proximal part of the graft was secured using whip stitches. The anterior and posterior borders of the LCL were isolated, and vascular clamps were passed under the LCL. Afterward, the free end of the ITB strip was slipped under the LCL from the proximal to the distal end. This maneuver is usually done with a vascular clamp or artery forceps. Special care is taken to remain in the central part of the ITB to preserve the Kaplan fibers, which are at a risk of damage if the posterior border of the ITB is incised. It should be noted that this step can be performed right after diagnostic arthroscopy, before har- vesting the ACL graft. Prolonged knee arthros- copy may lead to the swelling of the knee joint tissue, which can subsequently complicate tissue dissection and identification of the LCL. The fi- nal ITB strip fixation takes place after success- ful completion of the ACLR.

**FIGURE 3:**
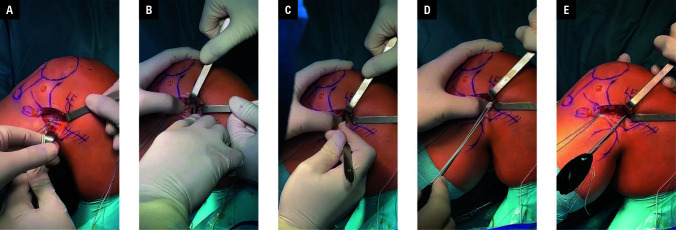
Intraoperative images showing the process of preparing soft tissues for anchor implantation; A–C – identification of the lateral epicondyle (LE), dissection of the remaining tissue using a vascular clamp without a 15 surgical blade, and exposition of the femoral cortex; D–F – placement of the anchor 20–30 degrees from the posterior to the anterior cortex of the femur (axial plane) and 20 degrees from the anterior part of the LE of the femur to the femoral canal (coronal plane) to avoid tunnel convergence

**TABLE 1:** Comparison of pre‑ and postoperative functional indicators

Questionnaire	Before surgery	After surgery	P value
KOOS‑pain	59.88 (19.26)	80.46 (15.52)	<0.001
KOOS‑symptoms	60.59 (23.78)	82.38 (15.72)	<0.001
KOOS‑ADL	67.39 (22.74)	90.36 (10.81)	<0.001
KOOS‑sport activity	36.95 (27.35)	70.47 (24.96)	<0.001
KOOS‑QoL	35.09 (22.09)	61.57 (18.23)	<0.001
IKDC	49.93 (19.59)	72.18 (15.32)	<0.001
Lysholm	49.47 (20.78)	77.31 (18.34)	<0.001
WOMAC	30.19 (20.08)	12.63 (12.08)	<0.001

Data are presented as mean (SD).

Abbreviations: ADL, activities of daily living; IKDC, International Knee Documentation Committee; KOOS, Knee Injury and Osteoarthritis Outcome Score; QoL, quality of life; WOMAC, Western Ontario and McMaster Universities Arthritis Index

After passing the ITB strip under the LCL, the lateral epicondyle (LE) was identified. The surrounding soft tissue around the femoral cortex was carefully dissected using vascular clamps, a surgical blade, or a finger. Of note, using finger dissection and palpation is a good method to avoid convergence with the ACLR button. The exact point of anchor was proximal and posterior to the LE FIGURE 3. Additionally, to prevent tunnel convergence, the anchor was positioned precisely 20–30 degrees from the posterior to the anterior cortex of the femur (axial plane) and 20 degrees from the anterior portion of the lateral condyle of femur (LCF) to the fem- oral canal (coronal plane). Drilling and locating a pilot hole is not necessary for self-punching all-suture anchors.^18^ The ITB strip was whip stitched using both wires that emerged from the suture anchor, and it was then drawn in the direction of the femur cortex. Subsequently, the graft was fixed to the LCF using a self-punching all-suture anchor (Linvatec Y-Knot RC 2.8 mm, CONMED) at 30 degrees of knee flexion in neutral rotation FIGURE 3.

During fixation, moderate tension of the ITB strip is sufficient, and maximum tension should be avoided. Knee range of motion should be assessed and cannot be restricted. Before closure, careful hemostasia was performed to minimize the risk of hematoma. In our opinion, the defect in the ITB should be obligatorily closed. The edges of the ITB were approximated using a continuous suture. We believe that suturing of the ITB additionally strengthens the anterolateral capsule. The subcutaneous layer was closed using a single suture (Ethicon). The skin closure is determined by the surgeon’s preference, utilizing either staples or standard sutures.

### Rehabilitation protocol

For every patient, a standard ACL rehabilitation program was followed. It included progressive range-of-motion exer- cises, an external hinge brace for 6 weeks, and partial weight-bearing with crutches following the treatment when there was no increased dis- comfort or effusion. The restricted range of mo- tion during the first 3 weeks was between 0 and 90 degrees, and between 90 and 120 degrees in the following 3 weeks. After 3 weeks, complete weight-bearing was permitted, whereas 6 weeks following the surgery, full active range of motion was allowed. Restoring active knee exten- sion and quadriceps activation was addressed in early therapy. Four months postsurgery, if there was no knee effusion and quadriceps strength was deemed sufficient, a progressive return to athletic activities was permitted. This included running on flat terrain and participation in non-pivoting sports. Athletes could return to pivoting noncontact sports approximately 6 months after the procedure, while the return to pivoting full-contact sports was typically allowed 8 to 9 months postoperation. The rehabilitation protocol was identical to that followed in the case of isolated ACLR.

**FIGURE 4:**
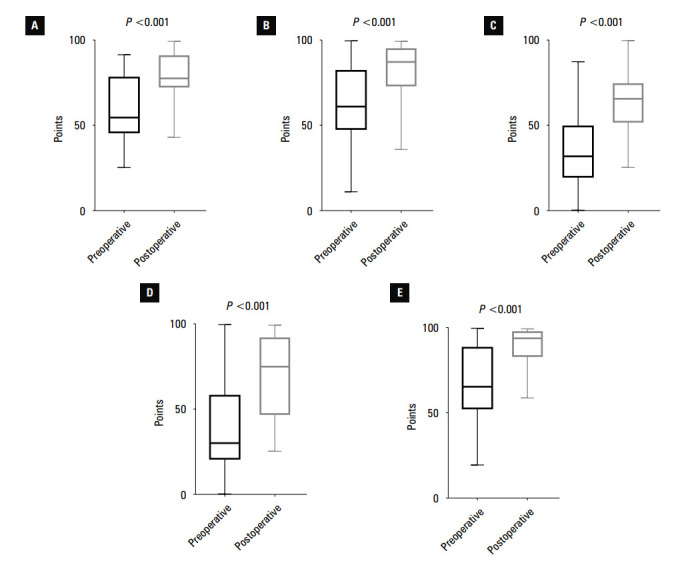
Boxplots showing pre- and postoperative Knee Injury and Osteoarthritis Outcome Score (KOOS) values; A – KOOS-pain; B – KOOS-symptoms; C – KOOS-quality of life; D – KOOS-sport activity; E – KOOS-activities of daily living. Horizontal lines represent the median, boxes represent the interquartile range, and whiskers represent minimal and maximal values.

### Follow‑up examination

All individuals under- went physical examination by 2 independent orthopedic surgeons who work together at the same unit (JZ and JE). The following clinical tests were performed: the Lahman test, the anterior drawer test, the posterior drawer test, the McMurray test, and the pivot shift test. The functional outcomes were measured using several questionnaires: the Knee Injury and Osteoarthritis Outcome Score (KOOS), assessing various domains (pain, symptoms, activities of daily living [ADL], sport activity, and quality of life), the International Knee Documentation Committee subjective knee evaluation form (IKDC), the Lysholm knee scoring scale, and the Western Ontario and McMaster Universities Arthritis Index (WOMAC). During the follow-up period, possible intra and postoperative com- plications or recurrence of injury were observed.

### Statystical analysis

Statistical analyses and group comparisons were conducted by 2 independent investigators using the GraphPad Prism software (GraphPad 8.0.1, Dotmatics, Boston, Massachusetts, United States). Nominal variables were de- scribed by the frequency of observations. Quan- titative data were summarized using descriptive statistics, including mean and SD for normally distributed data, and median and range for data with a non-normal distribution. Visual assess- ment of histograms was also performed. Their dis- tributions were confirmed using the Shapiro–Wilk test. The values of the questionnaires were com- pared using the Mann–Whitney test. A P value of less than 0.05 was considered significant.

## RESULTS

In this study, we analyzed 32 individ- uals who underwent concomitant ACL and LET procedures. The study population included 4 wom- en (12.5%) and 28 men (87.5%). Their mean (SD) age was 35.7 (12.1) years, ranging from 20 to 63 years. Five patients (15.6%) underwent revision ACL and LET surgery due to failure of the primary ACLR. Of the 20 patients (62.5%) diagnosed with additional meniscus tears, 15 had medial meniscus injury, 2 had lateral meniscus tears, and 3 had both. These were complex cases. According to the modified Outerbridge classification, grade 2 cartilage lesions were discovered in 7 patients and grade 3 in 1, whereas 26 patients (75%) showed no signs of cartilage damage. Clinical examinations were conducted after a mean (SD) 234.5 (136.3) days following surgery (range, 55–478 days).

**FIGURE 5:**
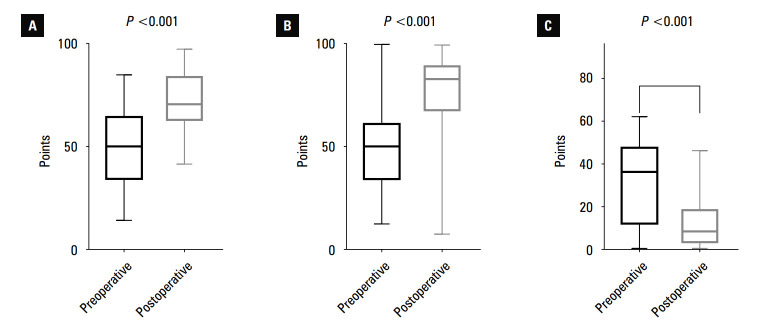
Example caption for this image

All patients had negative results of clinical tests, such as the Lahman, the posterior draw- er, and the McMurray tests; however, following surgery, 4 patients (12.5%) had a positive pivot shift test result (grade 1), and 1 patient (3.1%) had a positive anterior drawer test result (grade 1). Nonetheless, individuals with a grade 1 pivot shift or a grade 1 anterior drawer test result did not report any knee joint instability. There were no signs of reinjury or intra- or postoperative complications.

In all cases, the functional score values in- creased in comparison with the preoperative assessments. Increases in KOOS-pain, KOOS-symptoms, KOOS-ADL, KOOS-sport, IKDC, and the Lysholm scales were significant. Additionally, WOMAC ratings significantly decreased af- ter the surgery, indicating improved function of the afflicted lower limb. The outcomes are pre- sented in TABLE 1 and FIGURE 4 and FIGURE 5.

## DISCUSSION

The described novel mini-open technique of LET using a self-punching all-suture anchor was associated with improved clinical out- comes, minimal risk of complications, and smaller scarring, as compared with other LET techniques. The Lemaire technique was first described in 1967, and it was proposed as a stand-alone sur- gical treatment for ACL tear. Clinical outcomes were, however, poor. The primary technique

used a 15-cm incision and a 15 cm × 1.5 cm graft. The evolution of the procedure led to a reduc- tion in both the surgical incision length (7–8 cm) and the graft size (7–8 cm × 1 cm).^19^^,^^20^ The technique was renamed “modified Lemaire technique” and has since become one of the preferred methods among the LET techniques with concurrent ACLR.19, 21 However, the method of attaching the ITB strip to the femoral cortex remains a subject of debate, and its fixation depends on the surgeon, who may opt for staplers, interfer- ence screws, or anchors. For instance, a study by Behrendt et al^22^ compared anchor and interference screw fixation of the ITB graft with con- comitant revision ACLR, but did not find any significant clinical differences at 12-month follow-up. Our mini-open technique constitutes an ex- tension of the modified Lemaire technique with concurrent ACLR and recommends using a self-punching all-sutures anchor. The fundamentals of this technique are similar to those of the minimally invasive modified Lemaire technique de- scribed by Muller et al,^15^ but they differ in terms of surgical aspects and the hardware used. How- ever, both involve a 3–4 cm skin incision at the same location.

In our study, the LET procedure did not cause intra- or early postoperative complications. Heard et al^23^ observed that while there was no discernible rise in the incidence of other complications over a follow-up period of 2 years, the inclusion of LET during ACLR was linked to a decrease in ACL graft rupture and an increase in hard- ware irritation rates. They employed the modi- fied Lemaire technique using staples. The compli- cations related to LET were as follows: need to re- move LET hardware in 10 patients (3%), 3 intra- operative staple failures (1%), 3 postoperative he- matomas over the LET site (<1%), 2 cases of ITB snapping (<1%), and 1 case of over-constrained lateral compartment (<1%). Eggeling et al^24^ also described medical adverse events after LET pro- cedure using modified Lemaire technique. They found that 4 out of 23 patients who underwent revision ACLR experienced pain over the LET site 2 years after the procedure. The fixation of the ITB graft was performed using an interference screw attached to a 5-mm tunnel wired 1 cm proximal and posterior to the LE. The low complication rate after LET using the modified Lemaire technique was also reported by Declerq et al.^25^ The hardware they used was the SwiveLock anchor (Arthrex, Naples, Florida, United States) which required prior drilling of the tunnel. They noticed a single case of hematoma formation at the LET site and a single case of hardware irritation among 42 patients who were reviewed at a mean of 67.7 months. Notably, Behrendt et al^22^ compared an- chor and interference screw fixation of LET dur- ing the modified Lemaire technique among 52 patients undergoing revision ACLR. They report- ed 1 clinical failure in the anchor group without describing the details. The remaining complica- tions typically linked with LET augmentation per- formed using different techniques (ie, MacIntosh, Cocker–Arnold, Ellison) are local infections asso- ciated with LET hardware or sutures, damage to the LCL, and intraoperative rupture of the ITB graft.^12^^,^ ^26^^,^^27^^,^^28^.

Furthermore, a cadaveric study by Jaecker et al^29^ reported that the modified Lemaire technique may lead to tunnel convergence when combined with ACLR. In their re- search, the ITB strip was attached to a previously prepared 6 mm × 20 mm tunnel using an interference screw. In our study, tunnel convergence was not observed intraoperatively. Our technique seems to minimize the risk of its occurrence, since the self-punching all-suture anchor does not re- quire drilling, and its 2.8-mm footprint provides shallow but solid placement. Apart from the size of the implant used, another reason for the lack of tunnel conflict in our study is that our method recommends drilling the tunnel at a 20–30-degree angle in the axial plane and a 20-degree angle in the coronal plane. The studies on cadavers and humans showed that the drilling angle of at least 30 degrees in the axial plane decreased the risk of tunnel convergence nearly to zero.^30^^,^^31^ It is worth noting that hardware irritation (pain or discomfort), a relatively frequent complication in the abovementioned studies which sometimes warrants hardware removal, was not reported among our patients. The anchor used had a low profile that reduced hardware prominence, com- pared with staples or screws, which might ex- plain the lack of these particular adverse events. 

Primary or revision ACLR is a procedure frequently performed among middle-aged patients with chronic pain and knee instability. Indica- tions for additional LET procedures differ among studies and are based on poor-quality evidence. However, Getgood et al3^32^suggest the following criteria: revision ACLR, high-grade pivot shift, generalized ligamentous laxity, and young pa- tients participating in pivoting sports. Studies show that ACLR with LET augmentation restores knee function partially or fully, and results in improved postoperative clinical outcomes, as assessed by knee questionnaires (KOOS, IKDC, Lysholm, etc.).^33^ The extent of clinical improvement does not differ regardless of the graft used (hamstring, BTB, or quadriceps graft).^34^ Some studies recorded better functional scores and a higher rate of return to the preinjury sports performance in revision ACLR with LET augmentation, compared with revision ACLR alone, which may explain the abovementioned indications for additional LET procedure in revision ACLR.^35^^,^^36^^,^^37^^,^^38^ It is estimated that 63% of patients after a stand-alone ACLR restore their preinjury sports performance level.^39^ This study also showed a reduction in the severity of symptoms, reflected by higher scores in the KOOS, IKDC, and Lysholm scales, and lower WOMAC scores, regardless of primary or revision ACLR with LET augmentation.

This study has several limitations. There was no control group, and the sample size was relatively small, consisting of the local population with a combination of concomitant knee lesions. Com- plex injuries affect and complicate the recovery, rehabilitation protocol, and postoperative clinical assessments. However, other studies comprised a comparable number of participants with coexistent injuries. The follow-up period was different among the patients, and the time interval between the surgery and the examination was broad. Finally, all surgeries were performed by a single orthopedic surgeon, which could impact clinical outcomes. On the other hand, they were per- formed by a well-trained surgeon and instructor.

## CONCLUSIONS

This is the first study that fo- cused on the description and evaluation of clini- cal outcomes and complications after LET using the mini-open modified Lemaire technique in con- junction with ACLR. The reported technique us- ing a self-punching all-suture anchor appears to be an effective and safe approach that minimiz- es the risk of complications. It provides a short- er skin incision, which, compared with other LET techniques, is associated with better esthetics and does not require additional surgical devices and interventions, such as drilling of tunnels.
